# Incorporation of parental phenotypic data into multi‐omic models improves prediction of yield‐related traits in hybrid rice

**DOI:** 10.1111/pbi.13458

**Published:** 2020-09-02

**Authors:** Yang Xu, Yue Zhao, Xin Wang, Ying Ma, Pengcheng Li, Zefeng Yang, Xuecai Zhang, Chenwu Xu, Shizhong Xu

**Affiliations:** ^1^ Jiangsu Key Laboratory of Crop Genetics and Physiology Key Laboratory of Plant Functional Genomics of Ministry of Education Jiangsu Key Laboratory of Crop Genomics and Molecular Breeding Co‐Innovation Center for Modern Production Technology of Grain Crops Agricultural College of Yangzhou University Yangzhou China; ^2^ Department of Botany and Plant Sciences University of California Riverside CA USA; ^3^ International Maize and Wheat Improvement Center (CIMMYT) Mexico DF Mexico

**Keywords:** genomic selection, hybrid rice, multi‐omic data, parental traits, best linear unbiased prediction

## Abstract

Hybrid breeding has been shown to effectively increase rice productivity. However, identifying desirable hybrids out of numerous potential combinations is a daunting challenge. Genomic selection holds great promise for accelerating hybrid breeding by enabling early selection before phenotypes are measured. With the recent advances in multi‐omic technologies, hybrid prediction based on transcriptomic and metabolomic data has received increasing attention. However, the current omic‐based hybrid prediction has ignored parental phenotypic information, which is of fundamental importance in plant breeding. In this study, we integrated parental phenotypic information into various multi‐omic prediction models applied in hybrid breeding of rice and compared the predictabilities of 15 combinations from four sets of predictors from the parents, that is genome, transcriptome, metabolome and phenome. The predictability for each combination was evaluated using the best linear unbiased prediction and a modified fast HAT method. We found significant interactions between predictors and traits in predictability, but joint prediction with various combinations of the predictors significantly improved predictability relative to prediction of any single source omic data for each trait investigated. Incorporation of parental phenotypic data into various omic predictors increased the predictability, averagely by 13.6%, 54.5%, 19.9% and 8.3%, for grain yield, number of tillers per plant, number of grains per panicle and 1000 grain weight, respectively. Among nine models of incorporating parental traits, the AD‐All model was the most effective one. This novel strategy of incorporating parental phenotypic data into multi‐omic prediction is expected to improve hybrid breeding progress, especially with the development of high‐throughput phenotyping technologies.

## Introduction

Hybrid breeding facilitates crop production by taking advantage of heterosis. Hybrid rice and maize are the most successful cases that have greatly mitigated the global food crisis. However, finding the desirable hybrids from all potential crosses is a tremendous challenge in hybrid breeding. It is practically impossible to evaluate the performance of all potential hybrids in multi‐year and multi‐location trials due to limited resources. Genomic selection (GS) has been proposed as a promising strategy to confront these challenges. GS uses genome‐wide DNA markers and phenotypes from a training sample to predict the genetic values of candidates in a test sample, where the latter have been genotyped but not phenotyped (Crossa *et al*., 2017). As a result, GS enables early selection before phenotypes of traits are collected (Hickey *et al*., 2017). In hybrid breeding, GS is more effective because genotypes of hybrids are deduced from genotypes of their parents rather than sequenced anew, which has greatly reduced the cost of hybrid breeding (Xu *et al*., 2014). Several simulation and experimental studies have confirmed that GS is effective for hybrid prediction (Cui *et al*., 2020; Zhao *et al*., 2015). A GS model is often judged by its predictability, which is mainly calculated as the squared correlation coefficient between the predicted and observed phenotypic values. High predictability is a prerequisite for a successful application of GS. However, predictability is affected by many factors, including genetic architecture and heritability of traits, marker density, sample size, genetic diversity of the training sample, the relationship between training and test samples, and statistical models (Desta and Ortiz, 2014; Guo *et al*., 2019). Many researchers have been struggling to improve the predictability for some complex traits like grain yield in rice, but with little success. This may be due to the fact that GS has limitations in capturing the effects of gene interactions and downstream regulations (Ritchie *et al*., 2015).

Fortunately, downstream omes such as transcriptome, proteome and metabolome can capture interactions within and between different biological strata (Westhues and Schrag, 2017). With the rapid development in molecular technologies, transcriptomic and metabolomic information has been available for prediction (Li *et al*., 2018). Frisch *et al*. (2010) predicted hybrid performance and heterosis in maize based on transcription profiles from parental lines and concluded that the transcriptome‐based prediction outperforms genomic prediction. Based on the same data set, Fu *et al*. (2012) further assessed the accuracy of four different models in predicting maize hybrid performance using parental transcriptomic data. Zenke‐Philippi *et al*. (2017) found that transcription profiles are good alternatives to DNA markers for trait prediction under a ridge regression model. In addition to transcriptomic prediction, metabolomic prediction has also attracted attention from the GS community. By crossing 285 inbred lines with two testers, Riedelsheimer *et al*. (2012) predicted combining abilities for biomass‐related traits and found that the predictability with 130 metabolites is nearly as efficient as that with 56 110 SNPs. When parental metabolomic data were used to predict rice hybrid performance, the predictability of yield from a training sample of 278 hybrids is almost doubled compared with that of genomic prediction (Xu *et al*., 2016). Dan *et al*. (2016) reported high predictabilities of metabolomic prediction for plant height, heading date and grain yield based on a complete diallel cross experiment derived from 18 rice inbred lines. These studies suggest that both transcriptomic and metabolomic data are effective predictors for hybrid prediction.

Recently, there has been great interest in integrating multi‐omic data into a single model for trait prediction. Westhues and Schrag (2017) demonstrated the benefit of combining transcriptomic and genomic data in predicting important agronomic traits of hybrid maize. By comparing predictabilities from all combinations of three omic data using eight conventional prediction methods, Wang *et al*. (2019) concluded that the combination of genomic and metabolomic data generally provides the best prediction in rice. Despite the progress in hybrid prediction based on omic data, it remains a challenge regarding how to maximize the predictability from multiple sources of omic data. Previous omic prediction for hybrid performance mainly focused on genomic, transcriptomic and metabolomic data, but overlooked the phenotypic information of parents (phenome). In fact, phenotypes are the core of crop breeding; experienced breeders can, to some degree, guess the performance of hybrids based on the phenotypes of their parents (Furbank *et al*., 2019). Although previous studies showed that hybrid prediction solely based on the performance of parental lines per se is not as good as genomic prediction (Guo *et al*., 2013; Zhao *et al*., 2015), it remains unclear whether integrating parental phenotypic information into an omic prediction model can improve hybrid prediction.

Various statistical models have been developed for prediction, including the best linear unbiased prediction (BLUP), the least absolute shrinkage selection operator (LASSO), Bayesian methods, random forest, neural networks, support vector machine (SVM), and reproducing kernel Hilbert spaces regression (RKHS). Several studies have compared these prediction methods with empirical and simulated data (Xu *et al*., 2018; Xu *et al*., 2017). Although there is no method that is globally best for all data, the BLUP method often performs better than most other methods with high computational efficiency. As a result, BLUP has been a routine method for hybrid prediction. The predictability of BLUP is often evaluated by a K‐fold cross‐validation (CV), where the sample is randomly divided into *K* equal parts and each part is predicted once based on parameters estimated from the other *K*—1 parts. In a K‐fold CV, the predictability depends on the number of folds and the ways the sample is partitioned. To reduce the variability due to sample partitioning, researchers usually perform multiple rounds of K‐fold CV with different ways of sample partitioning and report the mean predictability over multiple runs. The leave‐one‐out‐cross‐validation (LOOCV, also called *n*‐fold CV), a special case of K‐fold CV with *K* equal to the number of observations (*n*), can be used to quantify the predictability without random errors due to sample partitioning (Cheng *et al*., 2017). However, LOOCV has a high computational cost for large data sets since the model has to be fit *n* times. Xu (2017) developed a HAT method to evaluate the predictive performance of BLUP, avoiding a lengthy process of CV analysis and greatly increasing the computational efficiency. Compared with a single omic prediction, multi‐omic prediction takes extended computing time with multiple kinship matrices. Therefore, multi‐omic prediction benefits the most from the HAT method.

The objectives of this work are to (1) modify the original HAT method for multi‐omic prediction and verify its feasibility, (2) evaluate the performance of hybrid prediction based on all combinations of the four sets of predictors including genomic data (G), transcriptomic data (T), metabolomic data (M) and phenomic data (P) of the parents using BLUP with the modified HAT method, (3) test whether incorporating parental phenotypic data can improve hybrid prediction and (4) further investigate the optimum model for incorporating parental phenotypic data.

## Results

### Comparison of the HAT method with the CV method

**Figure 1:**
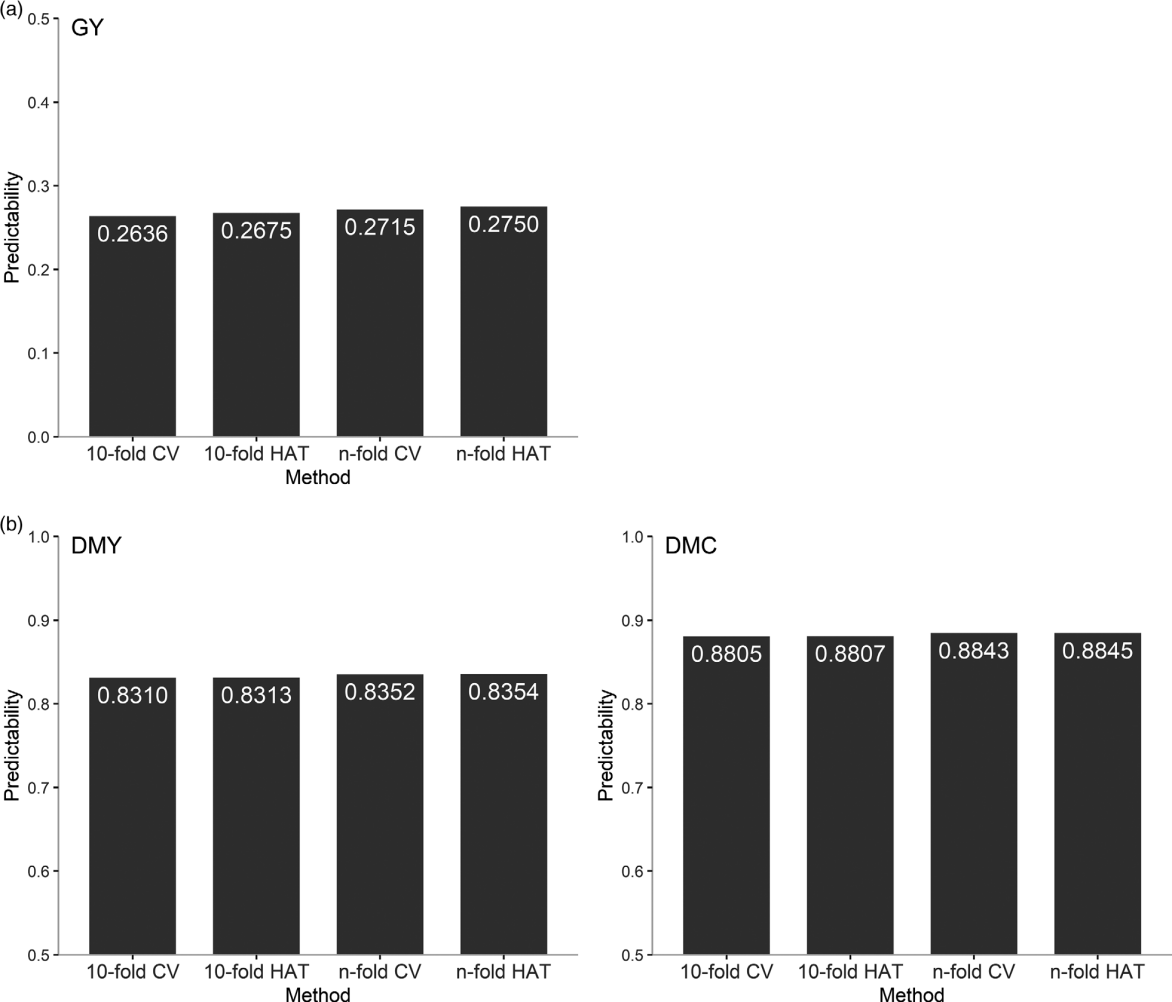
Comparison of predictabilities of the HAT and CV methods under 10‐fold and *n*‐fold. (a) Predictabilities of the HAT and CV methods for grain yield (GY) in wheat inbred lines. (b) Predictabilities of the HAT and CV methods for grain dry matter yield (DMY) and grain dry matter content (DMC) in maize hybrids. The predictabilities for 10‐fold HAT and 10‐fold CV were averaged over 50 replicates.

To demonstrate the suitability of the modified HAT method, we compared the HAT method with the CV method under 10‐fold and *n*‐fold using two publicly available data sets of wheat and maize. The wheat data set, available in the R package BGLR, consisted of 599 inbred lines genotyped with 1279 DArT (Diversity Array Technology) markers (Perez and de los Campos, 2014). The target trait was grain yield (GY). This data set was developed by the International Maize and Wheat Improvement Center (CIMMYT) as described previously (Crossa *et al*., 2010; Gianola *et al*., 2011). The predictabilities for the 10‐fold HAT and 10‐fold CV were averaged over 50 replicates. Regardless of the fold number, the HAT method and the CV method produce very similar predictabilities (Figure 1a). The difference between the 10‐fold HAT and the 10‐fold CV is 0.0039 and the difference between the *n*‐fold HAT and the *n*‐fold CV is 0.0035.

A similar comparison was also made in a maize population. The maize data set consisted of 550 hybrids from 50 Dent and 41 Flint inbred lines developed in a breeding program at the University of Hohenheim (Schrag *et al*., 2018). The hybrids were evaluated for grain dry matter yield (DMY) and grain dry matter content (DMC). Genomic data and metabolomic data were collected from parental lines. The genomic data contained 37 392 SNP markers and the metabolomic data contained 284 metabolites from roots measured 3.5 days after sowing. To test the effectiveness of the HAT method for multi‐omic prediction, we performed the prediction with the combination of genomic and metabolomic data. Figure 1b shows the predictabilities of DMY and DMC from the CV and HAT methods. Differences between the 10‐fold HAT and 10‐fold CV and those between the *n*‐fold HAT and *n*‐fold CV are barely noticed for both traits. Here, the *n*‐fold CV method took about ten hours to complete the calculations, while the *n*‐fold HAT method took approximately two minutes. Overall, for single omic prediction and multi‐omic prediction, the HAT method can be an excellent alternative to the CV method for evaluating predictability. In subsequent studies, we used the *n*‐fold HAT to evaluate the performance of hybrid prediction to avoid the long CV process and eliminate variation due to random partitioning of samples.

### Evaluation of predictabilities

**Figure 2:**
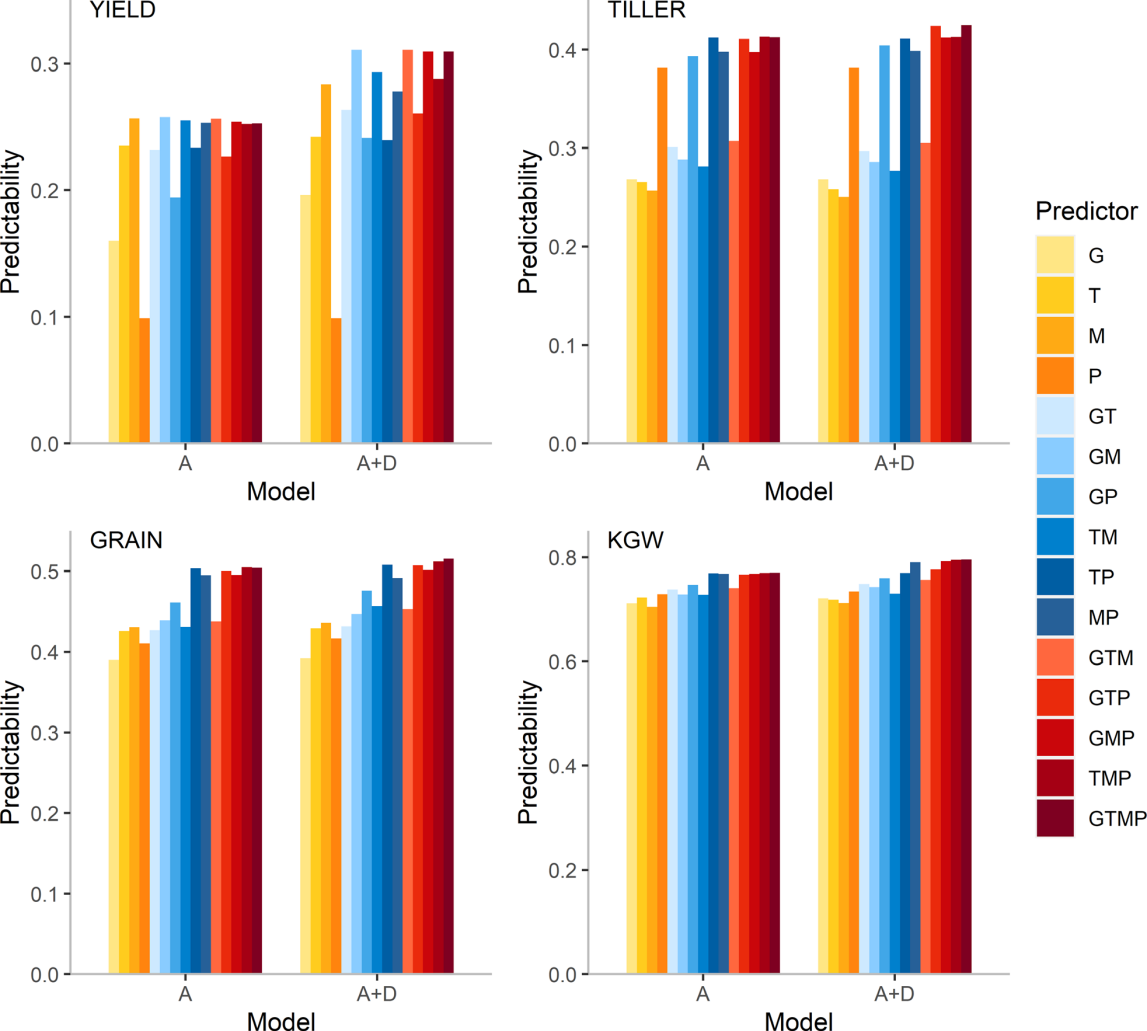
Predictabilities of four traits from 15 combinations of four predictors with the additive model and the additive‐dominance model. The four traits are YIELD, TILLER, GRAIN and KGW. The 15 predictor combinations are G, T, M, P, GT, GM, GP, TM, TP, MP, GTM, GTP, GMP, TMP and GTMP, where G, T, M and P represent genome, transcriptome, metabolome and phenome, respectively. ‘A’ denotes the additive model and ‘A + D’ denotes the additive‐dominance model. [Colour figure can be viewed at wileyonlinelibrary.com]

We evaluated the predictabilities of four traits using all 15 combinations of four sets of predictors (G, T, M, P, GT, GM, GP, TM, TP, MP, GTM, GTP, GMP, TMP and GTMP) together with the additive and additive‐dominance models (Figure 2), where G, T, M and P represent genome, transcriptome, metabolome and phenome, respectively. Among these four traits, 1000 grain weight (KGW) has the highest predictability, followed by number of grains per panicle (GRAIN), number of tillers per plant (TILLER) and lastly grain yield (YIELD). For YIELD, the additive‐dominance model exhibits a distinct advantage over the additive model, but for other traits, the advantage of the additive‐dominance model is not obvious. The differences in predictability among the predictors are small for high predictability traits and large for low predictability traits. For instance, the predictability varies from 0.10 to 0.31 for trait YIELD and from 0.70 to 0.79 for trait KGW. No single source of predictors achieves consistent superior predictability for all traits. For example, M is the best single source predictor for YIELD and GRAIN but is the worst predictor for TILLER and KGW, while P is the best single source of predictor for TILLER and KGW but the worst predictor for YIELD. Combining the best single source of predictors of an individual trait with other predictors can further improve predictability.

### Analysis of variance for predictabilities

**Figure 3:**
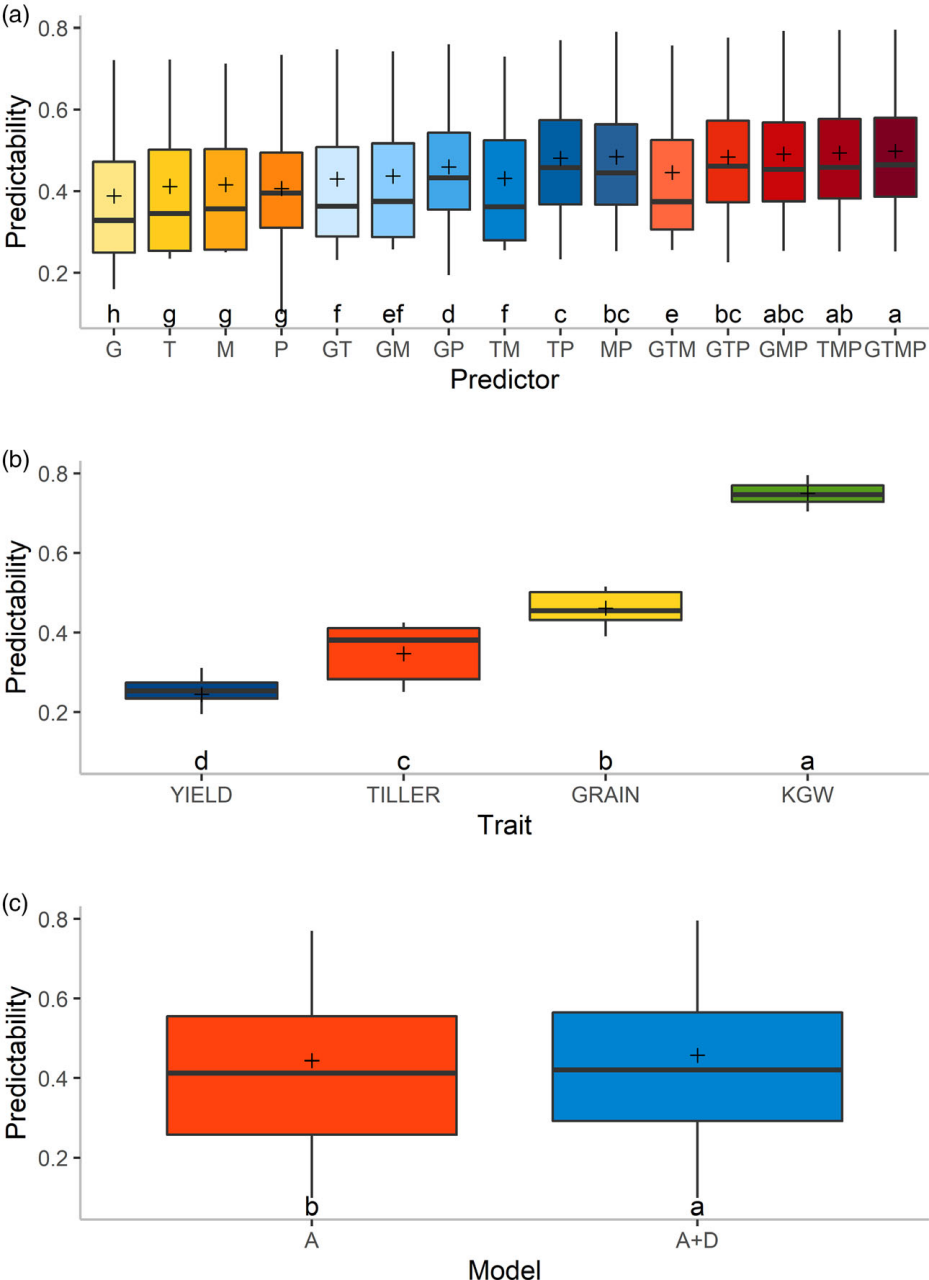
Multiple comparisons of predictabilities illustrated by boxplots. In each boxplot, different letters (in lower case) above the group labels indicate significant differences between groups. Positions of the plus sign represent the mean predictabilities. (a) Comparison of mean predictabilities of 15 predictors across four traits and two models. (b) Comparison of mean predictabilities for the four traits across 15 predictors and two models. (c) Comparison of mean predictabilities of the two models across 15 predictors and four traits. [Colour figure can be viewed at wileyonlinelibrary.com]

We performed an analysis of variance for the predictabilities of all 15 × 4×2 = 120 predictor‐trait‐model combinations. All the main effects and two‐way interaction effects are significant (Table S1). We then made multiple comparisons for significant main effects. The predictabilities of the 15 predictor combinations are classified into eight significant levels with GTMP being the best predictor and G being the worst predictor (Figure 3a). Overall, combined multiple predictors have significantly higher predictabilities than single predictors. For a single predictor, T, M and P predictors perform better than G. Among the combinations of two predictors, TP, MP and GP predictors are significantly superior over GT, GM and TM predictors. When three single predictors are combined, the GMP, TMP and GTP predictors outperform the GTM predictor. The results reveal that despite the single source of predictor P performs poorly, the predictor combinations comprising P are significantly better than those without P. Besides, the predictabilities of the four traits are significantly different. KGW is the best predictable trait and GY is the worst one (Figure 3b). Comparing the two models, the additive‐dominance model provides significantly higher predictability than the additive model (Figure 3c).

### Incorporating predictor P

**Figure 4:**
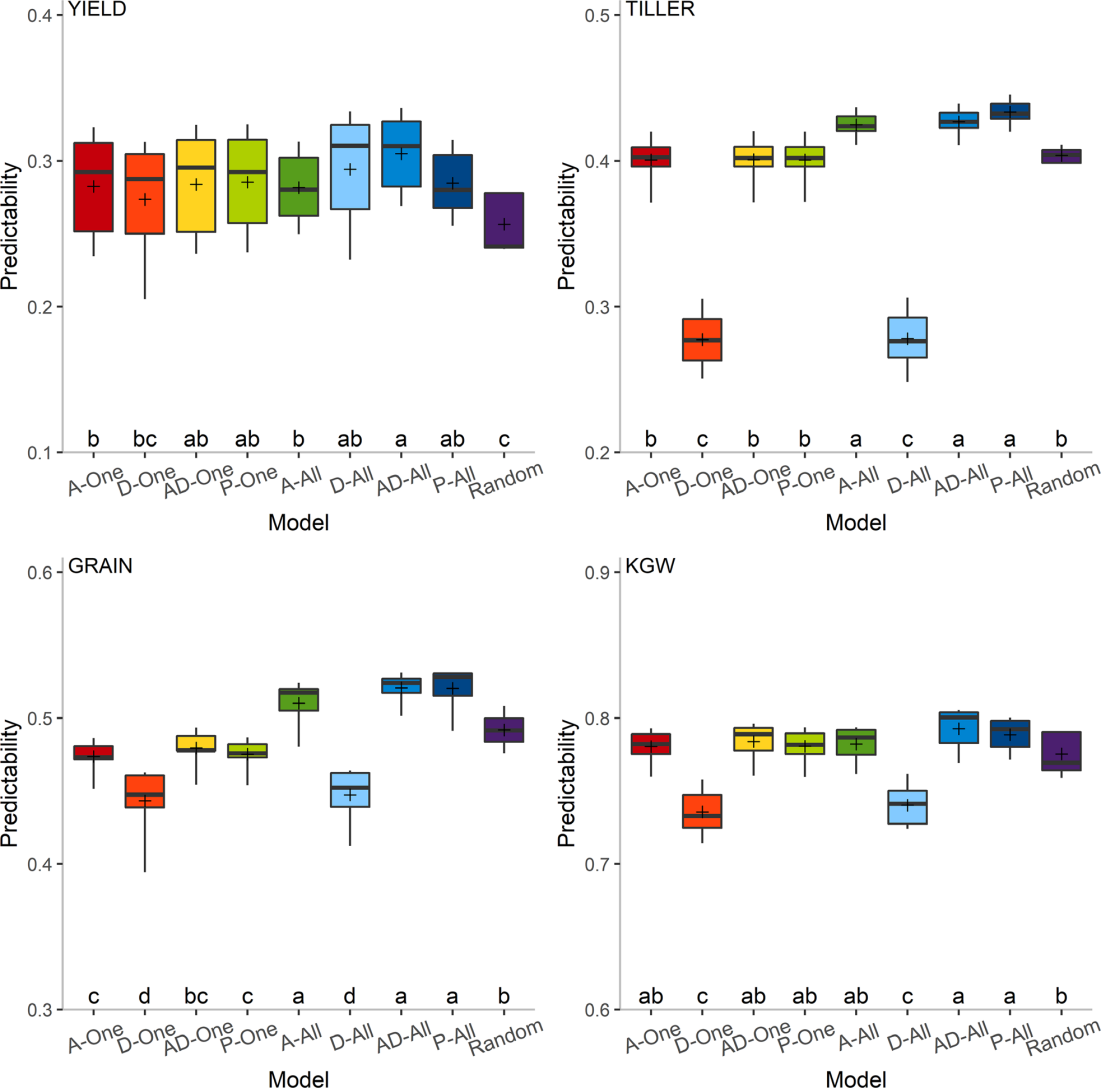
Multiple comparisons of mean predictabilities from nine models of incorporating predictor P across seven predictor combinations for four traits. The seven predictor combinations are GP, TP, MP, GTP, GMP, TMP and GTMP. [Colour figure can be viewed at wileyonlinelibrary.com]

According to the above studies, integrating predictor P with any other predictors has significantly improved the predictability. How to incorporate P into a current omic prediction model becomes an important issue. The parental phenotypic data are regarded as random variables so far in this study, while such data can also be regarded as fixed effects. A total of nine models (Random, A‐One, D‐One, AD‐One, P‐One, A‐All, D‐All, AD‐All, P‐All) of incorporating predictor P were compared. Here, the Random model treats the effects of predictor P as random effects, while the other models treat the effects of predictor P as fixed effects. In the A‐One, D‐One, AD‐One and P‐One models, we used parental phenotypic data just from the target trait, while in the A‐All, D‐All, AD‐All and P‐All models, we used parental phenotypic data from all traits. A detailed description of the models is provided in the Experimental procedures section. The predictabilities of the four traits from the seven predictor combinations containing predictor P (GP, TP, MP, GTP, GMP, TMP and GTMP) were calculated using the nine models (Figure S1). For each trait, there is a significant difference in the models (Table S2). The results of multiple comparisons for these nine models are depicted in Figure 4. For YIELD, the AD‐All model is the best and the Random model is the worst in terms of predictability. For GRAIN, TILLER and KGW, the AD‐All and P‐All models have the highest predictabilities, while the D‐One and D‐All models have the lowest predictabilities. Overall, the AD‐All model is the best model for incorporating predictor P.

**Figure 5:**
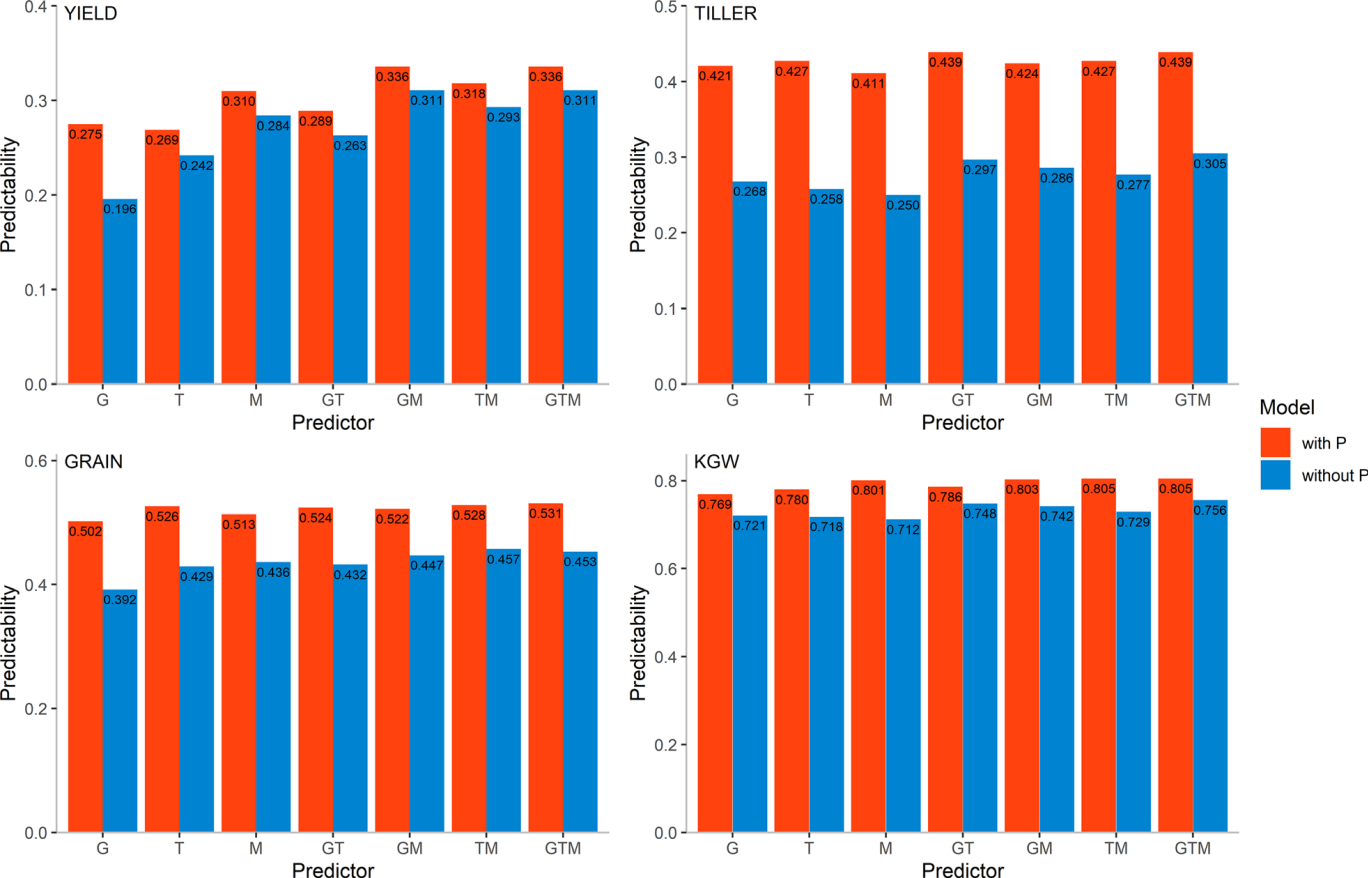
Comparison of predictabilities from the predictor combinations with predictor P (red) and the combinations without predictor P (blue) using the AD‐All model to incorporate predictor P. The predictor combinations with P are GP, TP, MP, GTP, GMP, TMP and GTMP, and the combinations without P are G, T, M, GT, GM, TM and GTM. [Colour figure can be viewed at wileyonlinelibrary.com]

Based on the AD‐All model, we integrated predictor P with other predictors and then evaluated their predictabilities. The predictabilities of the four traits from the predictor combinations with P and those without P are illustrated in Figure 5. In all traits, combining P with any other predictors increases the predictability, with the largest improvement occurring for TILLER. For YIELD, TILLER, GRAIN and KGW, the predictabilities with G, T, M, GT, GM, TM and GTM increase on average by 13.6%, 54.5%, 19.9% and 8.3%, respectively, when predictor P is added. The highest gains in predictability for YIELD (40.3%), TILLER (65.5%), GRAIN (28.1%) and KGW (12.5%) are obtained by integrating P into G, T, G and M, respectively.

### Predicting untested crosses

The training sample consisting of 278 hybrids represents a small subset of all 21 945 crosses derived from 210 recombinant inbred lines (RILs). Based on the parameters estimated from the training sample, we predicted all potential crosses for the four traits. Due to the low predictability of predictor P, we did not use predictor P alone but combined predictor P with other predictors to predict hybrids with the AD‐All model. The predicted phenotypic values of the 21 945 crosses from 14 predictors are listed in Data S1. Subsequently, the predicted phenotypic values were sorted in descending order. The means and standard deviations of predicted phenotypic values of the top 200 and bottom 200 selected crosses are displayed in Table 1. The average predicted values of the top 200 crosses are far higher than those of the bottom 200 crosses for all predictors of each trait. For example, when predictor GTMP is used, the average predicted values of the top 200 selection for YIELD, TILLER, GRAIN and KGW have been increased by 46.4%, 40.6%, 90.7%, 41.4%, respectively, compared with the average predicted phenotypic values of the bottom 200. Compared to the predictor combinations without P, the predictor combinations with P provide higher predicted values for the top 200 crosses and lower predicted values for the bottom 200 crosses. The predicted values for YIELD of the top 200 and the bottom 200 crosses are 49.3 and 37.6 on average across the seven predictor combinations without P, while the corresponding values across the predictor combinations with P are 53.3 and 34.5. Theoretically, the selected top crosses using the predictor combinations with P are expected to achieve a higher gain than those without P.

**Table 1:** Average predictabilities of four traits for the top 200 and bottom 200 crosses selected from all 21 945 potential crosses using 14 predictor combinations

Predictor	YIELD		TILLER		GRAIN		KGW	
	Top 200	Bottom 200	Top 200	Bottom 200	Top 200	Bottom 200	Top 200	Bottom 200
G	47.61 ± 0.39	39.24 ± 0.50	17.25 ± 0.11	13.94 ± 0.09	135.19 ± 1.65	95.81 ± 1.50	27.79 ± 0.15	22.49 ± 0.21
T	49.67 ± 0.87	37.63 ± 0.21	17.30 ± 0.12	13.61 ± 0.20	138.81 ± 0.65	92.20 ± 2.17	27.60 ± 0.10	22.19 ± 0.13
M	49.91 ± 0.65	37.06 ± 0.56	17.31 ± 0.19	13.83 ± 0.14	139.38 ± 1.82	89.22 ± 2.90	27.93 ± 0.27	22.09 ± 0.28
GT	49.18 ± 1.17	37.83 ± 0.25	17.34 ± 0.18	13.61 ± 0.16	137.39 ± 0.79	91.82 ± 1.77	27.80 ± 0.18	22.22 ± 0.21
GM	49.46 ± 0.70	37.16 ± 0.47	17.37 ± 0.13	13.91 ± 0.09	137.19 ± 1.48	90.77 ± 2.01	27.59 ± 0.14	22.30 ± 0.22
TM	49.99 ± 0.57	37.26 ± 0.46	17.35 ± 0.14	13.62 ± 0.19	139.36 ± 1.73	90.02 ± 3.01	27.63 ± 0.16	22.11 ± 0.23
GTM	49.46 ± 0.70	37.16 ± 0.47	17.38 ± 0.15	13.63 ± 0.14	137.58 ± 1.49	90.87 ± 2.26	27.67 ± 0.16	22.19 ± 0.22
GP	52.58 ± 1.21	35.11 ± 0.76	17.97 ± 0.25	12.99 ± 0.36	152.82 ± 5.32	80.42 ± 3.12	29.20 ± 0.41	20.72 ± 0.48
TP	54.69 ± 1.68	33.67 ± 0.93	18.18 ± 0.25	12.95 ± 0.27	156.29 ± 5.89	77.43 ± 3.84	29.20 ± 0.41	20.80 ± 0.46
MP	54.91 ± 1.81	33.39 ± 1.04	17.92 ± 0.24	13.04 ± 0.33	158.77 ± 6.61	76.38 ± 3.77	29.15 ± 0.42	20.84 ± 0.47
GTP	51.94 ± 1.11	35.50 ± 0.74	18.18 ± 0.25	12.92 ± 0.27	152.11 ± 4.96	80.47 ± 3.45	29.30 ± 0.42	20.63 ± 0.48
GMP	51.86 ± 1.15	35.42 ± 0.79	17.95 ± 0.25	12.98 ± 0.34	154.32 ± 5.61	79.50 ± 3.35	29.32 ± 0.43	20.59 ± 0.50
TMP	55.36 ± 1.88	33.11 ± 1.04	18.16 ± 0.25	12.97 ± 0.27	156.13 ± 5.79	77.91 ± 3.81	29.17 ± 0.41	20.83 ± 0.46
GTMP	51.86 ± 1.15	35.42 ± 0.79	18.16 ± 0.25	12.92 ± 0.27	152.93 ± 5.07	80.18 ± 3.54	29.26 ± 0.42	20.69 ± 0.48

## Discussion

For the first time, we integrated parental phenotypic information (phenome) into current multi‐omic prediction models for hybrid breeding of rice. The traits incorporated so far are all agronomic traits. Advanced phenotype facilities allow investigators to measure phenotypes of thousands of traits simultaneously. The biological functions of many such traits may not be known, but they can be used collectively to predict agronomic traits, just like SNPs of genome. If the predictor includes a large array of phenotypes, the method is called phenomic prediction. Although hybrid prediction from the phenotypes of inbred parents alone is not very effective (Smith, 1986), we found that combining parental phenotypic data with other predictors considerably improves hybrid prediction. Although P is a poor predictor for YIELD, as expected from the literature (Guo *et al*., 2013; Schrag *et al*., 2009), the predictor GP compared with G increases the predictability by up to 41%. These gains may be attributed to additional genetic information and genotype–environment interactions intrinsically captured by the phenotypic data (phenome). Therefore, parental phenotypic information should not be ignored in hybrid prediction. We also compared the predictive performances of 15 predictor combinations for hybrid prediction and found a significant interaction for predictabilities between predictors and traits. Among the single source predictors, the predictor M provides the best prediction for YIELD and GRAIN but the worst prediction for TILLER and KGW. Such interactions between predictors and traits have also been reported previously (Guo *et al*., 2016; Schrag *et al*., 2018). Although no single source of predictors preforms the best universally across all traits, combinations of these predictors provide consistently higher predictabilities than any of the components. The predictor combination GTMP is superior over the other combinations for all traits. The downstream predictors are expected to capture complex upstream interactions, so it is assumed that the downstream predictors can be a supplement to the upstream predictors. Essentially, genetic information flows from the genome to the phenotype via transcriptome, proteome and metabolome. To confirm the above assumption, we used genomic data as a baseline for prediction and then successively added transcriptomic, metabolomic and phenotypic data (i.e. predictors G, GT, GTM and GTMP). Figure S2 shows that the predictabilities for all traits increase from G to GTMP in turn. For example, for YIELD, the predictabilities of GT, GTM and GTMP increase by 38.9%, 61.6% and 69.7%, respectively, compared with G. A similar trend is also observed for other traits. Evidently, hybrid prediction benefits from the complementation of these predictors.

Despite the benefits of combining multiple predictors for hybrid prediction, the costs of obtaining omic data should not be ignored in practical breeding. Therefore, it is necessary to balance the improvement of predictability and cost. Results from this study show the benefits of incorporating parental phenotypic data for hybrid prediction. Combining genomic data and parental phenotypic data seems to be a cost‐effective choice for hybrid prediction. Although the joint predictor GTMP has the highest predictability, some problems exist in practical use of transcriptome and metabolome. Generally, transcript and metabolite profiles are dynamic and susceptible to environmental variation. A particular challenge in prediction based on transcriptomic and metabolomic data is the selection of appropriate tissue and sampling time (Westhues and Schrag, 2017). Since metabolites and transcripts suffer from measurement errors, multiple replicates are needed to neutralize these errors and this will substantially increase the cost. Furthermore, transcriptomic and metabolomic data are not collected from hybrids, but indirectly inferred from their parents. Unlike genomic data, the relationship between the transcript and metabolite levels of parents with those of hybrids is not clear, which may limit the application of such data.

How to effectively incorporate parental phenotypic information into the current prediction model is also an important issue. We used nine different models to add predictor P and compared their predictive performances. The result reveals that the AD‐All and P‐All models possess overall good performance (Figure 4). The AD‐All model incorporates both the mid‐parent value and the parental difference. In parent breeding, the parents selection should generate a population that meets the criterion of usefulness (Utz *et al*., 2001). Usefulness takes into consideration both the progeny mean and the genetic variance. Prediction of the progeny mean is often based on the mid‐parent value. Phenotypic and genetic distances between parents have been used to predict the genetic variance of progeny (Yao *et al*., 2018). Obviously, both the mid‐parent value and the parental difference are important factors for parental selection in hybrid breeding. In addition, the coding system for the AD‐All model matches the coding for the additive plus dominance model in genomic prediction. Since the P‐All model uses the original parental phenotypic values as predictors, the relationship between the hybrids and their parents can be directly estimated in the training population. This model allows the two parents to have different effects on the progeny, which may capture some of the maternal effects from the female parent (cytoplasmic inheritance). In general, both the AD‐All and P‐All models are viable choices for incorporating predictor P. We also found that using parental phenotypic data of all traits is more effective than using the target trait alone. This may be contributed by the consideration of the genetic and residual correlations among multiple traits. By using genomic data and multiple auxiliary traits of hybrids to predict target traits, Wang *et al*. (2017) found that the average predictabilities of the multi‐trait model with two auxiliary traits and eight auxiliary traits are 6.4% and 26.7% higher than those of the single‐trait model, respectively. In the present study, only four traits of parental lines were collected as predictor P. If phenotypes of more traits are used for prediction, the predictability is likely to increase. Such a phenomic prediction represents a new direction of hybrid breeding and further study is needed.

Recently, several statistical models of genomic prediction have been extended to multi‐omic prediction, including BLUP, random forest and SVM (Acharjee *et al*., 2016; Fu *et al*., 2012; Hu *et al*., 2019). However, overfitting problems arise when thousands of variables are trained over a relatively small sample. BLUP seems to be the most effective approach in handling multi‐omic data and is least influenced by overfitting compared with LASSO, PLS, SVM and SSVS (Wang *et al*., 2019). The BLUP method estimates the polygenic variance rather than the effect of each variable. Therefore, the computational efficiency of BLUP largely relies on the number of variance components and sample size. For the combined model with both additive and dominant effects, multiple variance components need to be estimated, which has considerably increased the computing time of BLUP. We have demonstrated the effectiveness of the fast HAT method by using two publicly available data sets of wheat and maize. We also compared the predictabilities of the HAT method with CV under *n*‐fold and 10‐fold for all predictor combinations with the additive model (Table S3). Although the predictabilities obtained from the HAT method are slightly higher than those of the CV method, the Pearson correlation coefficients (*r*) between the 10‐fold CV and 10‐fold HAT and between *n*‐fold CV and *n*‐fold HAT for all the four traits are approximately equal to 1, indicating very strong consistency between the two methods. The computational speed of the *n*‐fold HAT method is approximately *n* times faster than that of *n*‐fold CV. To reduce the computation burden, a modified multi‐omic HAT method can be used instead of the lengthy CV to measure the predictability of BLUP. Here, we focused on the HAT method for BLUP, and we will try to modify this method for other prediction models like LASSO and Bayesian methods. Previously, Gianola and Schön (2016) extended a similar approach to evaluating the predictability of RKHS and Bayesian methods, but they did not validate its effectiveness in multi‐omic prediction.

Our findings have successfully demonstrated the distinct benefit of incorporating parental phenotypic information for hybrid prediction in rice breeding. This novel strategy of incorporating parental phenotype coupled with the continuous development of high‐throughput phenotyping platforms and prediction models are expected to improve hybrid breeding progress not only for rice but also for other crop species with hybrid breeding as the main production mechanism.

## Experimental procedures

### Rice data set

The rice population of 360 crosses was generated from three rounds of pairing of 240 recombinant inbred lines (RILs) derived from a cross between Zhenshan 97 and Minghui 63. The mating design and genetic characteristics of the population were described in detail previously (Hua *et al*., 2003; Hua *et al*., 2002). Only 210 of the 240 RILs were available in the genomic, transcriptomic, metabolomic and phenotypic data. Totally, 278 hybrids derived from the 210 RILs were used in hybrid prediction. The phenotypic data of grain yield (YIELD), number of tillers per plant (TILLER), number of grains per panicle (GRAIN) and 1000 grain weight (KGW) of the 278 hybrids and their parents were collected from the experimental farm at Huazhong Agricultural University in 1998 and 1999 (Hua *et al*., 2003). Each year, the field experiment followed a randomized complete block design with two replicates. For each replicate, eight plants from every cross were sampled and the average trait values were treated as the original data. The average phenotypic values of the four replications were used for data analysis. Genomic, transcriptomic and metabolomic data were only collected from the 210 RILs. The genomic data were represented by 1619 recombinant bins inferred from 270 820 high‐quality SNPs identified in the rice genome (Xie *et al*., 2010; Yu *et al*., 2011). The transcriptomic data with 24 994 gene expression traits were quantified from the flag leaves. For each RIL, flag leaves were randomly selected from three plants at the heading stage in 2008 with two biological replicates, and the RNA samples of the two replicates were pooled for expression profiling (Wang *et al*., 2014). The metabolomic data contained 317 metabolites detected from germinated seeds and 683 metabolites detected from flag leaves harvested at the heading dates. Seeds from 15 seedlings per line were harvested in 2009 and 2010 with one biological replicate each year. In addition, flag leaves from three random plants per line were harvested at the heading dates in 2009 with two biological replicates (Gong *et al*., 2013). The original transcriptomic and metabolomic data were log2‐transformed for further analysis.

### Coding the hybrid predictors

Let $M\;\text{= \textbackslash{}\{}\;M_{\mathrm{jk}}\text{\textbackslash{}\}}\;$ and $F\;\text{= \textbackslash{}\{}\;F_{\mathrm{jk}}\text{\textbackslash{}\}}\;$ be n×m predictor matrices for the male and female parents of the corresponding hybrids, respectively, where *n* is the sample size (*n* = 210 for RILs and *n* = 278 for hybrids) and *m* is the number of independent variables for each set of predictors in the model (*m* = 1619, 24 994, 1000 and 4 for G, T, M and P, respectively). For the genomic data, the numerical code of marker *k* (*k* = 1, 2,…, *m*) for individual *j* (*j* = 1, 2,…, *n*) is defined as $M_{\mathrm{jk}}=F_{\mathrm{jk}}=1$ for the homozygote of the major allele *A*_1_, $M_{\mathrm{jk}}=F_{\mathrm{jk}}=0$ for the heterozygote $A_{1}A_{2}$, and $M_{\mathrm{jk}}=F_{\mathrm{jk}}=‐1$ for the homozygote of the minor allele *A*_2_. The additive genotype of the hybrid is defined as$Z_{\mathrm{jk}}=\frac{1}{2}\left(M_{\mathrm{jk}}+F_{\mathrm{jk}}\right)$, and the dominance genotype is defined as $W_{\mathrm{jk}}=\frac{1}{2}\left|M_{\mathrm{jk}}‐F_{\mathrm{jk}}\right|$. Assume that the genotypes of Zhenshan 97 and Minghui 63 are $A_{1}A_{1}$ and $A_{2}A_{2}$, respectively. The corresponding hybrid predictors are defined as

(1) $$Z_{\mathrm{jk}}=\left\{\begin{array}{c} 1=\frac{1}{2}\left(1+1\right)\\ 0=\frac{1}{2}\left(1‐1\right)\\ ‐1=‐\frac{1}{2}\left(1+1\right) \end{array}\begin{array}{ccc} & \text{for} & A_{1}A_{1}\\ & \text{for} & A_{1}A_{2}\\ & \text{for} & A_{2}A_{2} \end{array}\right)\;\;\;\;\;\;\;W_{\mathrm{jk}}=\left\{\begin{array}{ccc} 0=\frac{1}{2}\left|1‐1\right|\;\;\;\;\;\;\; & \text{for} & A_{1}A_{1}\\ 1\;=\frac{1}{2}\left|1‐\left(‐1\right)\right|\;\;\; & \text{for} & A_{1}A_{2}\\ 0=\frac{1}{2}\left|‐1‐\left(‐1\right)\right| & \text{for} & A_{2}A_{2} \end{array}\right)$$

The coding for transcriptomic, metabolomic and phenotypic data is consistent with the coding for genomic data. The additive coding of T, M and P is defined as $Z_{\mathrm{jk}}=\frac{1}{2}\left(M_{\mathrm{jk}}+F_{\mathrm{jk}}\right)$, and the dominance coding is defined as $W_{\mathrm{jk}}=\frac{1}{2}\left|M_{\mathrm{jk}}‐F_{\mathrm{jk}}\right|$, where $M_{\mathrm{jk}}$ and $F_{\mathrm{jk}}$ represent the measurements of the corresponding predictor (T, M or P) for male and female parents, respectively. Details of coding system were described in our previous study (Xu *et al*., 2016). These predictors were standardized prior to the data analysis.

### Hybrid prediction model

The hybrid prediction model based on a single source of predictors is defined as

(2) $$y=X\beta +Z\gamma_{a}+W\gamma_{d}+\varepsilon$$

where y is an n×1 vector of the phenotypic values, *X* is an *n* × *q* design matrix for the fixed effect *β*, $Z\;\text{= \textbackslash{}\{}\;Z_{\mathrm{jk}}\text{\textbackslash{}\}}\;$ is an *n* × *m* design matrix for the additive effect $\gamma_{a}$ (*m* × 1 vector), $W\;\text{= \textbackslash{}\{}\;W_{\mathrm{jk}}\text{\textbackslash{}\}}\;$ is an *n* × *m* design matrix for the dominance effect $\gamma_{d}$, and ε is a vector of residual errors with an assumed $N(0,I\sigma^{2})$ distribution. Assume that $\gamma_{a}∼N\left(0,\frac{1}{m}\phi_{a}^{2}\right)$ and $\gamma_{d}∼N\left(0,\frac{1}{m}\phi_{d}^{2}\right)$, where $\phi_{a}^{2}$ and $\phi_{d}^{2}$ are polygenic variances of additive and dominance effects, respectively. The expectation of *y* is E(y)=Xβ and the variance–covariance matrix is

(3) $$\text{var}\left(y\right)=V=\frac{1}{m}ZZ^{T}\phi_{a}^{2}+\frac{1}{m}ZZ^{T}\phi_{d}^{2}+I\sigma^{2}=K_{a}\phi_{a}^{2}+K_{d}\phi_{d}^{2}+I\sigma^{2}$$

where *K*_a_ and *K*_d_ are kinship matrices for additive and dominance effects. The variance components $\left\{\phi_{a}^{2},\;\phi_{d}^{2},\;\sigma^{2}\right\}$ are estimated with the restricted maximum likelihood (REML) method. The whole sample is partitioned into a training sample and a test sample. Parameters estimated from the training sample are used to predict the phenotypic values of the test sample. Here, $y_{1}$ is an $n_{1}\times 1$ vector of phenotypic values for the training sample and $y_{2}$ is an $n_{2}\times 1$ vector of phenotypic values for the test sample. The partitioned model is written as

(4) $$\left[\begin{array}{c} y_{1}\\ y_{2} \end{array}\right]=\left[\begin{array}{c} X_{1}\beta \\ X_{2}\beta \end{array}\right]+\left[\begin{array}{c} Z_{1}\gamma_{a}\\ Z_{2}\gamma_{a} \end{array}\right]+\left[\begin{array}{c} Z_{1}\gamma_{d}\\ Z_{2}\gamma_{d} \end{array}\right]+\left[\begin{array}{c} \varepsilon_{1}\\ \varepsilon_{2} \end{array}\right]$$

and the variance–covariance matrix is partitioned accordingly as

(5) $$\text{var}\left[\begin{array}{c} y_{1}\\ y_{2} \end{array}\right]=\left[\begin{array}{cc} V_{11} & V_{12}\\ V_{21} & V_{22} \end{array}\right]=\left[\begin{array}{cc} K_{a11} & K_{a12}\\ K_{a21} & K_{a22} \end{array}\right]\phi_{a}^{2}+\left[\begin{array}{cc} K_{d11} & K_{d12}\\ K_{d21} & K_{d22} \end{array}\right]\phi_{d}^{2}+\left[\begin{array}{cc} I & 0\\ 0 & I \end{array}\right]\sigma^{2}$$

where $K_{a11}$ and $K_{d11}$ are the kinship matrices for the training sample, $K_{\text{a22}}$ and $K_{d22}$ are the kinship matrices for the test sample, and $K_{a21}$ and $K_{d21}$ are the kinship matrices between individuals in the test sample and individuals in the training sample. The predicted phenotypic values for the test sample is interpreted as the conditional expectation of $y_{2}$ given $y_{1}$, which is expressed as

(6) $${\hat{y}}_{2}=E\left(y_{2}\left|y_{1}\right)\right)=X_{2}\hat{\beta }+\left(K_{a21}{\hat{\phi }}_{a}^{2}+K_{d21}{\hat{\phi }}_{d}^{2}\right)V_{11}^{‐1}\left(y_{1}‐X_{1}\hat{\beta }\right)$$

The above prediction model includes additive and dominance effects, so it is called the additive‐dominance model. If only the additive effect is involved, the model becomes the additive model.

When using multiple predictors, the fully combined model is modified as

(7) $$y=X\beta +Z_{G}\gamma_{\mathrm{Ga}}+Z_{T}\gamma_{\text{Ta}}+Z_{M}\gamma_{\text{Ma}}+Z_{P}\gamma_{\text{Pa}}+W_{G}\gamma_{\text{Gd}}+W_{T}\gamma_{\text{Td}}+W_{M}\gamma_{\text{Md}}+W_{P}\gamma_{\text{Pd}}+\varepsilon$$

where $Z_{G}$, $Z_{T}$, $Z_{M}$ and $Z_{P}$ are the design matrices for the additive effects of G, T, M and P predictors, respectively; $W_{G}$, $W_{T}$, $W_{M}$ and $W_{P}$ are the design matrices for the dominance effects of the corresponding predictors. Let the polygenic variances of additive effects $\gamma_{\text{Ga}}$, $\gamma_{\text{Ta}}$, $\gamma_{\text{Ma}}$ and $\gamma_{\text{Pa}}$ be $\phi_{\text{Ga}}^{2}$, $\phi_{\text{Ta}}^{2}$, $\phi_{\text{Ma}}^{2}$ and $\phi_{\text{Pa}}^{2}$, respectively, and the polygenic dominance variances corresponding to the dominance effects $\gamma_{\text{Gd}}$, $\gamma_{\text{Td}}$, $\gamma_{\text{Md}}$ and $\gamma_{\text{Pd}}$ be $\phi_{\text{Gd}}^{2}$, $\phi_{\text{Td}}^{2}$, $\phi_{\text{Md}}^{2}$ and $\phi_{\text{Pd}}^{2}$, respectively. The expectation of *y* is E(y)=Xβ and the variance–covariance matrix is

(8) $$\text{var}\left(y\right)=V=K_{\text{Ga}}\phi_{\text{Ga}}^{2}+K_{\text{Ta}}\phi_{\text{Ta}}^{2}+K_{\text{Ma}}\phi_{\text{Ma}}^{2}+K_{\text{Pa}}\phi_{\text{Pa}}^{2}+K_{\text{Gd}}\phi_{\text{Gd}}^{2}+K_{\text{Td}}\phi_{\text{Td}}^{2}+K_{\text{Md}}\phi_{\text{Md}}^{2}+K_{\text{Pd}}\phi_{\text{Pd}}^{2}+I\sigma^{2}$$

The unknown parameters are estimated using the REML method whose likelihood function is

(9) $$L\left(\theta \right)=‐\frac{1}{2}\ln|V|‐\frac{1}{2}\ln\left|X^{T}V^{‐1}X\right|‐\frac{1}{2}{\left(y‐X\beta \right)}^{T}V^{‐1}\left(y‐X\beta \right)$$

Once the parameters are estimated based on the training sample, the prediction of the test sample follows the procedure described in equation (6) by integrating all variance components. Such a prediction model is called the full model. Reduced models include a subset of the four omic predictors.

### The HAT method for multi‐omic prediction

To simplify the argument, two predictors are considered in the multi‐omic prediction model. The prediction model with two predictors is reformulated as

(10) $$y=X\beta +\xi_{1}+\xi_{2}+\varepsilon$$

where y denotes a vector of the phenotypic values; Xβ represents the fixed effects; $\xi_{1}$ and $\xi_{2}$ are random effects for two sets of predictors with $\xi_{1}∼N\left(0,K_{1}\sigma_{\xi_{1}}^{2}\right)$ and $\xi_{2}∼N\left(0,K_{2}\sigma_{\xi_{2}}^{2}\right)$ distributions, respectively; and $\varepsilon ∼N(0,I\sigma^{2})$ is a vector of residual errors. The expectation of *y* is E(y)=Xβ and the variance–covariance matrix of *y* is $\text{var}\left(y\right)=V=K_{1}\sigma_{\xi_{1}}^{2}+K_{2}\sigma_{\xi_{2}}^{2}+I\sigma^{2}$, where *K*_1_ and *K*_2_ are kinship matrices for the two sets of predictors. The parameters $\left\{\beta ,\;\sigma_{\xi_{1}}^{2},\;\sigma_{\xi_{2}}^{2},\;\sigma^{2}\right\}$ are estimated with the REML method. Let $\xi \;=\;\xi_{1}+\xi_{2}=y‐X\hat{\beta }$ be the ‘observed’ effects for predictors (these are the phenotypic effects adjusted by the fixed effects), and let $\hat{\xi }\;=\;{\hat{\xi }}_{1}+{\hat{\xi }}_{2}=\hat{y}‐X\hat{\beta }$ be the predicted effects. By solving the mixed model equations, $\hat{\xi }$ based on the whole sample is derived as

(11) $$\hat{\xi }=\left(K_{1}{\hat{\sigma }}_{\xi_{1}}^{2}+K_{2}{\hat{\sigma }}_{\xi_{2}}^{2}\right)V^{‐1}\left(y‐X\hat{\beta }\right)=\left(K_{1}{\hat{\sigma }}_{\xi_{1}}^{2}+K_{2}{\hat{\sigma }}_{\xi_{2}}^{2}\right)V^{‐1}\xi$$

Corresponding to $\hat{\xi }=H\xi$, $H^{R}=\left(K_{1}{\hat{\sigma }}_{\xi_{1}}^{2}+K_{2}{\hat{\sigma }}_{\xi_{2}}^{2}\right)V^{‐1}$ should be the HAT matrix of the random effects. For the K*‐*fold HAT method, the sample needs to be partitioned into *K* parts. Let $\hat{e}=\xi_{k}‐{\hat{\xi }}_{k}$ be the estimated residual error for the sample in the *k*th part and $H_{\mathrm{kk}}^{R}$ be the diagonal block of 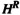 for the sample in the *k*th part (*k* = 1, 2,…, *K*). The predicted residual error for the *k*th part is $e_{k}={\left(I‐H_{\mathrm{kk}}^{R}\right)}^{‐1}{\hat{e}}_{k}$, and this has been proved in (Xu, 2017). Therefore, the predicted residual error sum of squares (PRESS) is expressed as.

(12) $$\text{PRESS}=\sum_{k=1}^{K}e_{k}^{T}e_{k}=\sum_{k=1}^{K}{\hat{e}}_{k}^{T}{\left(I‐H_{\mathrm{kk}}^{R}\right)}^{‐2}{\hat{e}}_{k}$$

The predictability is defined as

(13) $$R_{\text{HAT}}^{2}=1‐\text{PRESS}/\text{SS}$$

where SS is the total sum of squares of the *y* adjusted for the mean. $R_{\text{HAT}}^{2}$ is approximately equal to the commonly used predictability measured by the squared correlation coefficient between the predicted and observed phenotypic values (adjusted by the fixed effects). Similar to CV, the *n*‐fold HAT method is a particular case of K‐fold HAT with *K* = *n*. For the *n*‐fold HAT, PRESS is modified as.

(14) $$\text{PRESS}=\sum_{j=1}^{n}e_{j}^{2}=\sum_{j=1}^{n}{\hat{e}}_{j}^{2}{\left(I‐H_{\mathrm{jj}}\right)}^{‐2}$$

where $H_{\mathrm{jj}}$ is the *j*th diagonal element of the HAT matrix. To avoid sample partitioning errors, we used the *n*‐fold HAT method to evaluate the predictability in this study.

### Incorporating phenotypic information of parental lines

The phenotypic data of parental lines are treated as random variables in the prediction model described above, but such data can also be considered as fixed variables. To explore the full potential of parental phenotypic information, we incorporated the phenotypic data using various models and compared their predictive performances. These models include$(1)P=\frac{1}{2}(P_{M}+P_{F})\;\beta_{a},(2)P=\frac{1}{2}|P_{M}‐P_{F}|\beta_{d},(3)P=\frac{1}{2}(P_{M}+P_{F})\;\beta_{a}+\frac{1}{2}|P_{M}‐P_{F}|\beta_{d}and(4)P_{M}\beta_{M}+P_{F}\;\beta_{F}$, where *P* is a set of phenotypic predictors, $P_{M}$ and $P_{F}$ are the phenotypic data for the male and female parents of the corresponding hybrids; $\beta_{a}$, $\beta_{d}$, $\beta_{M}$ and $\beta_{F}$ are the model effects. These four models are called A, D, AD and P models, respectively. For the first three models, $\frac{1}{2}\left(P_{M}+P_{F}\right)$ represents the mid‐parent value and $\frac{1}{2}\left|P_{M}‐P_{F}\right|$ represents the difference between the two parents. Model (4), the P model, does not involve any recoding but simply takes the original phenotypic values of the parents as predictors. Additionally, the $P_{M}$ and $P_{F}$ can be the parental phenotypic data from all traits ($P_{M\;‐\;\text{All}}$ and $P_{F‐\text{All}}$) or just from the target trait ($P_{M‐\text{One}}$ and $P_{F‐\text{One}}$). Here, a total of nine scenarios (Random, A‐One, D‐One, AD‐One, P‐One, A‐All, D‐All, AD‐All, P‐All) were considered when combining the phenotypic data of parental lines. The fully combined models incorporating four sets of predictors are summarized in Table 2. All statistical analyses were performed using R software. The data and codes are available on GitHub (https://github.com/yangxu89/GS2020).

**Table 2:** The formula for nine different fully combined models of genomic, transcriptomic, metabolomic and phenotypic data

Model	Formula
Random	$y=X\beta +Z_{G}\gamma_{\text{Ga}}+Z_{T}\gamma_{\text{Ta}}+Z_{M}\gamma_{\text{Ma}}+Z_{P}\gamma_{\text{Pa}}+W_{G}\gamma_{\text{Gd}}+W_{T}\gamma_{\text{Td}}+W_{M}\gamma_{\text{Md}}+W_{P}\gamma_{\text{Pd}}+\varepsilon$
A‐One	$y=X\beta +\frac{1}{2}\left(P_{M\;‐\;\text{One}}+P_{F\;‐\;\text{One}}\right)\;\beta_{a}+Z_{G}\gamma_{\text{Ga}}+Z_{T}\gamma_{\text{Ta}}+Z_{M}\gamma_{\text{Ma}}+W_{G}\gamma_{\text{Gd}}+W_{T}\gamma_{\text{Td}}+W_{M}\gamma_{\text{Md}}+\varepsilon$
D‐One	$y=X\beta +\frac{1}{2}\left|P_{M\;‐\;\text{One}}‐P_{F\;‐\;\text{One}}\right|\beta_{d}+Z_{G}\gamma_{\text{Ga}}+Z_{T}\gamma_{\text{Ta}}+Z_{M}\gamma_{\text{Ma}}+W_{G}\gamma_{\text{Gd}}+W_{T}\gamma_{\text{Td}}+W_{M}\gamma_{\text{Md}}+\varepsilon$
AD‐One	$y=X\beta +\frac{1}{2}\left(P_{M\;‐\;\text{One}}+P_{F\;‐\;\text{One}}\right)\;\beta_{a}+\frac{1}{2}\left|P_{M\;‐\;\text{One}}‐P_{F\;‐\;\text{One}}\right|\beta_{d}+Z_{G}\gamma_{\text{Ga}}+Z_{T}\gamma_{\text{Ta}}+Z_{M}\gamma_{\text{Ma}}+W_{G}\gamma_{\text{Gd}}+W_{T}\gamma_{\text{Td}}+W_{M}\gamma_{\text{Md}}+\varepsilon$
P‐One	$y=X\beta +P_{M\;‐\;\text{One}}\beta_{M}+P_{F\;‐\;\text{One}}\;\beta_{F}+Z_{G}\gamma_{\text{Ga}}+Z_{T}\gamma_{\text{Ta}}+Z_{M}\gamma_{\text{Ma}}+W_{G}\gamma_{\text{Gd}}+W_{T}\gamma_{\text{Td}}+W_{M}\gamma_{\text{Md}}+\varepsilon$
A‐All	$y=X\beta +\frac{1}{2}\left(P_{M\;‐\;\text{All}}+P_{F\;‐\;\text{All}}\right)\;\beta_{a}+Z_{G}\gamma_{\text{Ga}}+Z_{T}\gamma_{\text{Ta}}+Z_{M}\gamma_{\text{Ma}}+W_{G}\gamma_{\text{Gd}}+W_{T}\gamma_{\text{Td}}+W_{M}\gamma_{\text{Md}}+\varepsilon$
D‐All	$y=X\beta +\frac{1}{2}\left|P_{M\;‐\;\text{All}}‐P_{F\;‐\;\text{All}}\right|\beta_{d}+Z_{G}\gamma_{\text{Ga}}+Z_{T}\gamma_{\text{Ta}}+Z_{M}\gamma_{\text{Ma}}+W_{G}\gamma_{\text{Gd}}+W_{T}\gamma_{\text{Td}}+W_{M}\gamma_{\text{Md}}+\varepsilon$
AD‐All	$y=X\beta +\frac{1}{2}\left(P_{M\;‐\;\text{All}}+P_{F\;‐\;\text{All}}\right)\;\beta_{a}+\frac{1}{2}\left|P_{M\;‐\;\text{All}}‐P_{F\;‐\;\text{All}}\right|\beta_{d}+Z_{G}\gamma_{\text{Ga}}+Z_{T}\gamma_{\text{Ta}}+Z_{M}\gamma_{\text{Ma}}+W_{G}\gamma_{\text{Gd}}+W_{T}\gamma_{\text{Td}}+W_{M}\gamma_{\text{Md}}+\varepsilon$
P‐All	$y=X\beta +P_{M\;‐\;\text{All}}\beta_{M}+P_{F\;‐\;\text{All}}\;\beta_{F}+Z_{G}\gamma_{\text{Ga}}+Z_{T}\gamma_{\text{Ta}}+Z_{M}\gamma_{\text{Ma}}+W_{G}\gamma_{\text{Gd}}+W_{T}\gamma_{\text{Td}}+W_{M}\gamma_{\text{Md}}+\varepsilon$

The nine models include one random model (Random) and eight fixed models (A‐One, D‐One, AD‐One, P‐One, A‐All, D‐All, AD‐All, P‐All). The random model treats parental phenotypic data as random variables and the fixed model treats parental phenotypic data as fixed variables.

## Conflict of interest

The authors declare no conflict of interest.

## Authors contributions

C.X., S.X. and Y.X. designed the research; Y.X., Z.Y. and X.Z. performed the research; Y.Z., X.W., Y.M. and P.L. analysed the data; and Y.X. wrote the paper. All authors read and approved the final manuscript.

## Supporting information


**Figure S1** Predictabilities of four traits from seven predictor combinations comprising the predictor P with nine models.
**Figure S2** Predictabilities of four predictors (G, GT, GTM, and GTMP) for four traits.
**Table S1** Analysis of variance of predictabilities from a 15×2×4 factorial design with 15 predictors, two prediction models, and four traits.
**Table S2** Analysis of variance of predictabilities from a 9×7 factorial design with nine models and seven predictors for four traits.
**Table S3** Predictabilities of the HAT and CV methods under 10‐fold and *n*‐fold for four traits from 15 predictor combinations.

**Data S1** Predicted four traits for all 21,945 potential crosses derived from 210 recombination inbred lines from 14 predictors.
