# Supplementary material for: Incorporation of parental phenotypic data into multi‐omic models improves prediction of yield‐related traits in hybrid rice
Source: Plant Biotechnol J. 2020 Sep 2;19(2):261–72. doi: 10.1111/pbi.13458 (PMC7868986; doi:10.1111/pbi.13458)
Supplement: Supplementary file 1 — Figure S1 Predictabilities of four traits from seven predictor combinations comprising the predictor P with nine models. Figure S2 Predictabilities of four predictors (G, GT, GTM, and GTMP) for four traits. Table S1 Analysis of variance of predictabilities from a 15×2×4 factorial design with 15 predictors, two prediction models, and four traits. Table S2 Analysis of variance of predictabilities from a 9×7 factorial design with nine models and seven predictors for four traits. Table S3 Predictabilities of the HAT and CV methods under 10‐fold and n‐fold for four traits from 15 predictor combinations. [file PBI-19-261-s001.pdf]

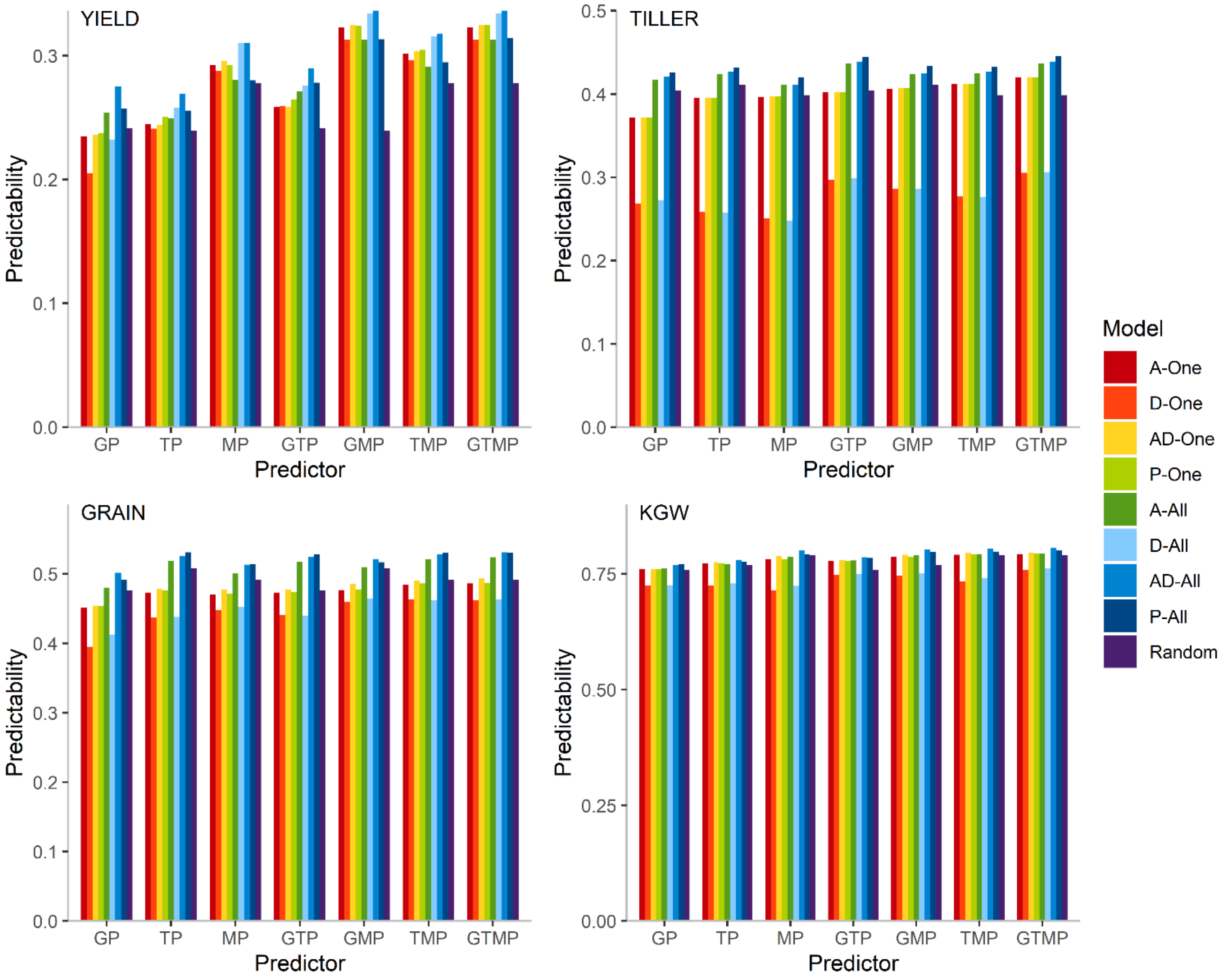

Figure S1. Predictabilities of four traits from seven predictor combinations comprising the predictor P with nine models. The seven predictor combinations are GP, TP, MP, GTP, GMP, TMP, and GTMP, where G, T, M, and P represent genome, transcriptome, metabolome, and phenome, respectively.

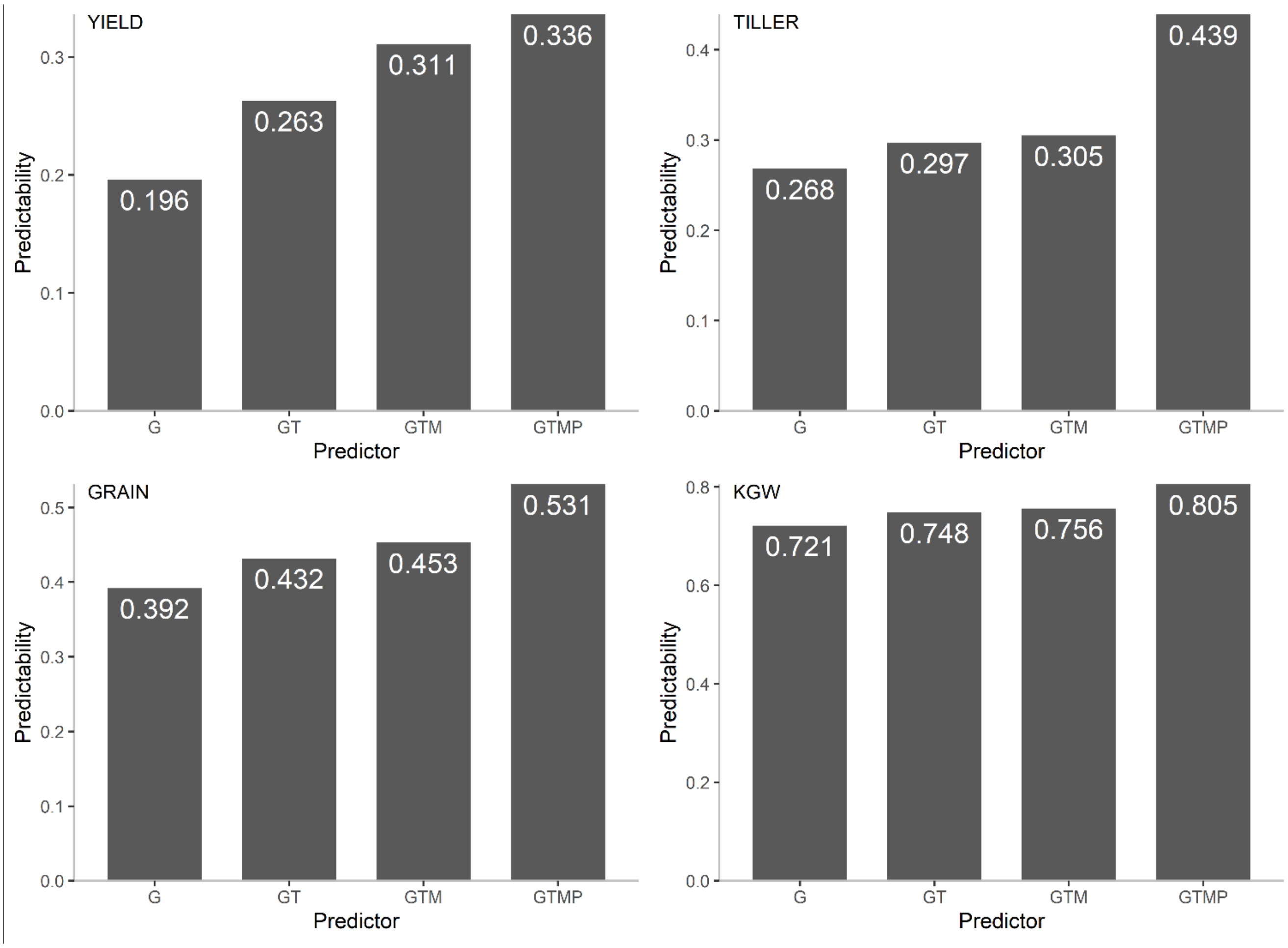

Figure S2. Predictabilities of four predictors (G, GT, GTM, and GTMP) for four traits.

Table S1. Analysis of variance of predictabilities from a 15×2×4 factorial design with 15 predictors, two prediction models, and four traits

Source of variation	DF	Sum of Squares	Mean Squares	F-test	*P*-value
Predictor	14	0.148	0.0106	240.312	<2e-16***
Model	1	0.006	0.0057	130.597	1.80e-14***
Trait	3	4.284	1.4279	32497.439	<2e-16***
Predictor: Model	14	0.002	0.0001	3.015	0.00285**
Predictor: Trait	42	0.111	0.0026	59.930	<2e-16***
Model: Trait	3	0.004	0.0015	33.312	3.49e-11***
Residuals	42	0.002	0.0000		

The 15 predictors are G, T, M, P, GT, GM, GP, TM, TP, MP, GTM, GTP, GMP, TMP, and GTMP. The two prediction models are additive model and additive-dominance model.

Table S2. Analysis of variance of predictabilities from a 9×7 factorial design with nine models and seven predictors for four traits

Trait	Source of variation	DF	Sum of Squares	Mean Squares	F-test	*P*-value
YIELD	Predictor	6	0.0504	0.0084	57.99	<2e-16 ***
	Model	8	0.0098	0.0012	8.496	4.1e-07 ***
	Residuals	48	0.0070	0.0001		
TILLER	Predictor	6	0.0068	0.0011	12.34	2.35e-08 ***
	Model	8	0.2083	0.0260	284.52	<2e-16 ***
	Residuals	48	0.0044	0.0001		
GRAIN	Predictor	6	0.0095	0.0016	25.95	1.67e-13 ***
	Model	8	0.0464	0.0058	94.9	<2e-16 ***
	Residuals	48	0.0029	0.0001		
KGW	Predictor	6	0.0072	0.0012	23.46	9.89e-13 ***
	Model	8	0.0240	0.0030	58.83	<2e-16 ***
	Residuals	48	0.0024	0.0001		

The seven predictor combinations are GP, TP, MP, GTP, GMP, TMP, and GTMP. The nine models are Random, A-One, D-One, AD-One, P-One, A-All, D-All, AD-All, P-All.

Table S3. Predictabilities of the HAT and CV methods under 10-fold and *n*-fold for four traits from 15 predictor combinations

Predictor	YIELD				TILLER				GRAIN				KGW			
	10-fold CV	10-fold HAT	*n*-fold CV	*n*-fold HAT	10-fold CV	10-fold HAT	*n*-fold CV	*n*-fold HAT	10-fold CV	10-fold HAT	*n*-fold CV	*n*-fold HAT	10-fold CV	10-fold HAT	*n*-fold CV	*n*-fold HAT
G	0.140	0.149	0.154	0.160	0.249	0.255	0.264	0.268	0.366	0.374	0.385	0.390	0.693	0.695	0.710	0.712
T	0.209	0.222	0.224	0.235	0.239	0.251	0.255	0.266	0.403	0.411	0.417	0.426	0.697	0.703	0.717	0.722
M	0.236	0.245	0.249	0.257	0.232	0.244	0.247	0.257	0.409	0.416	0.424	0.431	0.674	0.679	0.699	0.705
P	0.096	0.098	0.097	0.099	0.380	0.380	0.381	0.381	0.409	0.410	0.410	0.410	0.728	0.728	0.729	0.729
GT	0.201	0.219	0.218	0.232	0.277	0.289	0.291	0.301	0.403	0.413	0.417	0.427	0.714	0.720	0.733	0.737
GM	0.236	0.246	0.248	0.258	0.266	0.277	0.279	0.288	0.420	0.428	0.433	0.439	0.707	0.711	0.724	0.728
GP	0.181	0.187	0.189	0.194	0.385	0.391	0.388	0.393	0.451	0.456	0.457	0.461	0.739	0.743	0.743	0.746
TM	0.225	0.243	0.237	0.255	0.253	0.269	0.266	0.281	0.400	0.416	0.415	0.431	0.698	0.707	0.719	0.727
TP	0.204	0.222	0.220	0.233	0.402	0.410	0.405	0.412	0.489	0.497	0.497	0.504	0.753	0.762	0.764	0.769
MP	0.232	0.243	0.243	0.253	0.387	0.395	0.390	0.398	0.484	0.489	0.489	0.495	0.758	0.763	0.764	0.768
GTM	0.227	0.245	0.238	0.256	0.279	0.296	0.292	0.307	0.411	0.426	0.425	0.438	0.717	0.723	0.735	0.740
GTP	0.197	0.216	0.210	0.226	0.397	0.408	0.398	0.411	0.483	0.494	0.491	0.500	0.739	0.760	0.758	0.766
GMP	0.230	0.244	0.241	0.254	0.382	0.395	0.384	0.397	0.480	0.490	0.486	0.495	0.757	0.763	0.763	0.768
TMP	0.221	0.242	0.232	0.252	0.396	0.411	0.399	0.413	0.488	0.499	0.494	0.505	0.756	0.764	0.763	0.769
GTMP	0.220	0.243	0.229	0.253	0.392	0.410	0.392	0.412	0.484	0.498	0.490	0.504	0.756	0.765	0.763	0.770
r	0.993		0.994		0.998		0.997		0.996		0.995		0.988		0.996	

r indicates the Pearson correlation coefficients between 10-fold CV and 10-fold HAT and between *n*-fold CV and *n*-fold HAT.
Predictabilities for 10-fold HAT and 10-fold CV were averaged over 50 replicates.
